# Zona Glomerulosa–Derived Klotho Modulates Aldosterone Synthase Expression in Young Female Mice

**DOI:** 10.1210/endocr/bqae040

**Published:** 2024-04-04

**Authors:** Arezoo Daryadel, Cong Tang, Ye Xie, Mirko Peitzsch, Viktoria Fisi, Constanze Hantel, Dominique Loffing-Cueni, David T Breault, David Penton, Johannes Loffing, Felix Beuschlein

**Affiliations:** Department of Endocrinology, Diabetology and Clinical Nutrition, University Hospital of Zürich (USZ) and University of Zürich (UZH), 8091 Zurich, Switzerland; Department of Endocrinology, Diabetology and Clinical Nutrition, University Hospital of Zürich (USZ) and University of Zürich (UZH), 8091 Zurich, Switzerland; Department of Endocrinology, Diabetology and Clinical Nutrition, University Hospital of Zürich (USZ) and University of Zürich (UZH), 8091 Zurich, Switzerland; Institute of Clinical Chemistry and Laboratory Medicine, University Hospital and Medical Faculty Carl Gustav Carus, Technische Universität Dresden, 01307 Dresden, Germany; Department of Endocrinology, Diabetology and Clinical Nutrition, University Hospital of Zürich (USZ) and University of Zürich (UZH), 8091 Zurich, Switzerland; Department of Endocrinology, Diabetology and Clinical Nutrition, University Hospital of Zürich (USZ) and University of Zürich (UZH), 8091 Zurich, Switzerland; Institute of Anatomy, University of Zürich, 8057 Zurich, Switzerland; Division of Endocrinology, Department of Pediatrics, Boston Children's Hospital, Harvard Medical School, Boston, MA 02115, USA; Harvard Stem Cell Institute, Harvard University, Cambridge, MA 02138, USA; Electrophysiology Facility, University of Zurich, 8057 Zürich, Switzerland; Institute of Anatomy, University of Zürich, 8057 Zurich, Switzerland; Department of Endocrinology, Diabetology and Clinical Nutrition, University Hospital of Zürich (USZ) and University of Zürich (UZH), 8091 Zurich, Switzerland; Medizinische Klinik und Poliklinik IV, Klinikum der Universität, Ludwig-Maximilians-Universität, 81377 Munich, Germany; The LOOP Zurich Medical Research Center, 8044 Zurich, Switzerland

**Keywords:** Klotho, aldosterone, zona glomerulosa, CYP11B2, Cre-lox system

## Abstract

Klotho plays a critical role in the regulation of ion and fluid homeostasis. A previous study reported that haplo-insufficiency of Klotho in mice results in increased aldosterone synthase (*CYP11B2*) expression, elevated plasma aldosterone, and high blood pressure. This phenotype was presumed to be the result of diminished Klotho expression in zona glomerulosa (zG) cells of the adrenal cortex; however, systemic effects on adrenal aldosterone production could not be ruled out. To examine whether Klotho expressed in the zG is indeed a critical regulator of aldosterone synthesis, we generated a tamoxifen-inducible, zG-specific mouse model of Klotho deficiency by crossing *Klotho-flox* mice with *Cyp11b2-CreERT* mice (zG-Kl-KO). Tamoxifen-treated *Cyp11b2-CreERT* animals (zG-Cre) served as controls. *Rosa26-mTmG* reporter mice were used for Cre-dependent lineage-marking. Two weeks after tamoxifen induction, the specificity of the zG-Cre line was verified using immunofluorescence analysis to show that GFP expression was restricted to the zG. RNA in situ hybridization revealed a 65% downregulation of Klotho messenger RNA expression in the zG of zG-Kl-KO female mice at age 12 weeks compared to control mice. Despite this significant decrease, zG-Kl-KO mice exhibited no difference in plasma aldosterone levels. However, adrenal *CYP11B2* expression and the *CYP11B2* promotor regulatory transcription factors, NGFIB and Nurr1, were enhanced. Together with in vitro experiments, these results suggest that zG-derived Klotho modulates Cyp11b2 but does not evoke a systemic phenotype in young adult mice on a normal diet. Further studies are required to investigate the role of adrenal Klotho on aldosterone synthesis in aged animals.

Klotho has been identified as a gene associated with accelerating aging when disrupted and extending lifespan when overexpressed (1-4). Klotho is highly expressed in the kidney and has been implicated as a regulator of fluid and electrolyte balance that might also modulate aldosterone secretion from the zona glomerulosa (zG) of the adrenal cortex. On a molecular level, Klotho functions as a coreceptor of (fibroblast growth factor) FGF receptors to specifically activate the FGF23 signaling pathway (5, 6). Of interest, FGF23 may also be involved in the activation of the local renin-angiotensin-aldosterone system (RAAS), including hyperaldosteronism in cardiac fibrosis and hypertrophy progression (7, 8), chronic kidney disease (9, 10), diabetic nephropathy (11), and hepatic failure (12). Conversely, aldosterone has been recognized as a powerful stimulator of FGF23 formation in vitro and in vivo by enhancement of store-operated Ca^2+^ entry (13). Furthermore, the interplay of Klotho and the RAAS has been further demonstrated on the basis of downregulation of Klotho by angiotensin II (Ang II) and upregulation of cytochrome P450 (CYP) 11B2, which is responsible for aldosterone synthesis in the absence of Klotho (5, 6). Conversely, Klotho downregulates RAAS activity, thus attenuating tissue injury and fibrosis and improving hypertension in experimental chronic kidney disease (6). As adrenal zG cells are the primary source of circulating aldosterone and CYP11B2 is the specific and rate-limiting enzyme for aldosterone synthesis in zG cells, we aimed to address the question whether aldosterone production is modulated in a zG-depleted Klotho mouse model. To date, different in vivo studies had shown the interrelation of Klotho and CYP11B2 and aldosterone synthesis in different transgenic Klotho mouse models (14-16). In the present study, we explored the role of zG-derived Klotho on systemic aldosterone production in zG-Kl mice and human NCI-H295R cells as an in vitro model of hyperaldosteronism (17).

## Materials and Methods

### Generation of Transgenic Mice

#### *Cyp11b2* (aldosterone synthase)^+/Cre^–*Klotho^fl/fl^* mice

*Cyp11b2^+/Cre-ER^* mice (18) is a novel mouse line that allows transgenic modifications specifically in zG cells. It is designed to express the tamoxifen-inducible Cre transgene under the control of the zG specific transcript *Cyp11b2*. Conditional Klotho knockout (KO) mice were custom-generated by Ozgene (Bentley DC) by flanking exon 2 with loxP sites (manuscript in preparation). The *Cyp11b2^+/Cre-ER^* mice were cross-bred to *Klotho^fl/fl^* mice to generate zG-depleted *Klotho* mice (zG-Kl-KO) under tamoxifen (TMX)-induced Cre recombination. Heterozygosity at the *Cyp11b2* allele was maintained in all experimental animals including control mice (*Cyp11b2^+/Cre-ER—^Klotho^+/+^*) (zG-Cre).

#### *Cyp11b2^+/Cre-ER^–Klotho^fl/fl^*-*mTmG* mice

**Figure 1.**
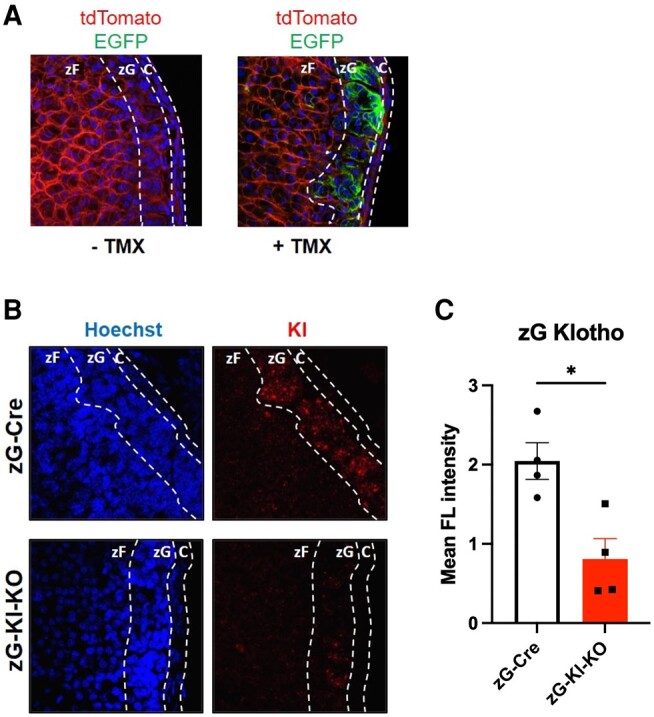
Klotho expression is diminished in the zona glomerulosa (zG) in zG-Kl-KO mice. A, Specificity of Cre activity was determined in 8-week-old female inducible *CYP11B2^+/Cre-ER^*-*mTmG* mice that received either vehicle or tamoxifen (TMX) (2 mg/kg/day) for 5 consecutive days. Following 2 weeks of recovery, the *CYP11B2-Cre-ER*–expressing cells were labeled with green fluorescence due to TMX-induced Cre activity. B, Klotho expression and depletion in the adrenal gland was determined by in situ hybridization in both zG-Kl-KO and zG-Cre animals, indicating specific expression in the zG. C, Following quantification by fluorescence intensity (FL) measurement, reduction of expression was evident in zG-Kl-KO animals. Stars represent significance vs corresponding control; *P less than or equal to .05.

*Cyp11b2^+/Cre-ER^*-*Klotho^fl/fl^* mice were crossed with *Gt(ROSA)26Sor^tm4(ACTB-tdTomato-EGFP)Luo/^J* reporter line (Jackson: 007676) to generate *Cyp11b2^+/Cre-ER^*-*Klotho^fl/fl^–mTmG*. The mice express membrane-targeted red fluorescence before and membrane-targeted green fluorescence after TMX induced Cre recombination (Fig. 1A).

To induce the Cre activity, all mice received the 2 mg/kg of TMX (Sigma; T5648) dissolved in sunflower seed oil (Sigma; S5007) daily by gavage for 5 consecutive days following 2 weeks of recovery before further study.

#### Metabolic cage studies

After induction of Cre activity, control (zG-Cre) or (zG-Kl-KO) female mice were fed constantly with standard diet (KLIBA NAFAG) and received water ad libitum. Animals were placed individually into metabolic cages (Tecniplast) for 3 days, and water and food intake as well as urine production and body weight were measured. Following 2 days of adaptation, urine was collected under mineral oil and stored at −20 °C for ion measurements. On day 3, the animals were anesthetized with isoflurane and venous blood was taken, centrifuged (8000 rpm, 10 minutes at 4 °C) and the plasma was stored at −80 °C. Then, adrenal and kidneys were collected and stored at −80 °C.

All procedures were carried out according to the Swiss animal welfare laws and guidelines for animal care and were approved by the Veterinary Office of the Canton Zurich (license No. 173/2019).

#### Adrenocortical cell line and primary culture

Human NCI-H295R cells originally from ATCC were cultured in a 1:1 mixture of Dulbecco’s modified Eagle’s medium (DMEM) and Ham's F12 medium (DMEM-F12 and supplements, Gibco Invitrogen). The medium was supplemented with insulin-transferrin selenium (ITS) solution at a final concentration of insulin 10 mg/L, transferrin 5.5 mg/L, and sodium-selenite 6.7 ng/L (ITS). Additionally, penicillin (100 U/mL), streptomycin (100 µg/mL), and 2% ultroser G (Cytogen) were added (19).

About 70% to 80% confluent NCI-H295R cells were stably transfected either with scramble *Klotho* guide RNA (gRNA) (CRISPR-Cas9 Negative Control crRNA #1, Integrated DNA Technologies, catalog No. 1072544) or a mixture of 2 *Klotho* gRNAs (protospacer sequence of gRNAs is TCCCCGTAGCACCTCACTGT and CCTAATGGAGACATTCATTT) using the NEON Transfection System following the manufactural protocol from Integrated DNA Technologies with 2-pulse 20 ms, 1300 V.

Additionally, the confluent NCI-H295R cells were seeded in 24-well plates at a concentration of 1 × 10^5^ cells/mL for 24 hours. Cells were then serum-starved overnight and subsequently incubated with 1 µM recombinant human Klotho (Sigma; SRP3102) following 30 minutes or 6 hours with 100 nM Ang II (Sigma; A9525), NaCL (9 mM; 12 mM), and KCL (9 mM; 12 mM) and cells were lysed for RNA and protein extraction.

Eight- to 12-week-old female C57BL/6 wild-type mice were anesthetized with isoflurane and adrenal tissues were removed and dissected under anesthesia. Each adrenal gland was cut into 4 pieces that were transferred into DMEM-F12 containing 3.6 mg/mL collagenase (Sigma; C6885) and were dissociated by MACS shaker for 40 minutes (program 37C_MALT_A_01). DMEM-F12 containing 10% fetal calf serum was added to stop the collagenase activity and single cells were collected via centrifugation at 500*g* for 5 minutes. Cells were incubated overnight with and without 1 µM recombinant human Klotho (Sigma; SRP3102) followed by stimulation of 100 nM Ang II (Sigma; A9525), NaCL (9 mM), and KCL (9 mM) for 6 and 24 hours and cells were lysed for RNA and protein extractions.

#### Determination of urinary and plasma electrolytes

Urinary and plasma levels of calcium, phosphate, potassium, magnesium, and creatinine were measured with a UniCel DxC 800 Synchron Clinical System (Beckman Coulter) in the Zurich Integrative Rodent Physiology (ZIRP) facility.

Plasma levels of adrenocorticotropin (ACTH) (MDB; M046006, RRID: AB_2893258) and plasma renin concentration were determined by enzyme-linked immunosorbent assay and radioimmunoassay techniques, respectively, according to the manufacturers’ protocols as described previously (20).

#### Determination of plasma steroids

For functional assessment of mouse steroid hormone levels, liquid chromatography–tandem mass spectrometry was performed as described previously (21).

#### RNA isolation and semiquantitative real-time polymerase chain reaction

RNA was isolated from mouse adrenal and kidney tissues, human NCI-H295R cells, and mouse primary adrenal cells using the Quick-RNA MiniPrep (ZYMO Research; R1055) according to instructions provided by the manufacturer. Purified RNA was transcribed to complementary DNA (TaqMan Reverse Transcription Kit, Applied Biosystems), which was later used as template for the polymerase chain reaction. The relative expression of the genes of interest was quantified using sequence-specific forward and reverse primers together with sequence-specific probes labeled with reporter (5'-end, FAM) and quencher (3'-end, TAMRA) dyes. Custom-designed primers and probes were synthetized by Microsynth, whereas commercially available primers were purchased from Applied Biosystems (Supplementary Tables 1 and 2 (22)). *Ppib* (peptidylpropyl isomerase B) and *HPRT* (hypoxanthine guanine phosphoribosyltransferase) were used as housekeeping genes. The relative fold change was calculated according to the formula 2^(Ct (gene of interest)− Ct(reference gene))^.

#### RNAscope in situ hybridization

Mouse adrenals were freshly harvested under anesthesia and processed for embedding (O.C.T. embedding matrix; Cell Path). Embedded and frozen adrenals were cut into 12-μm thick sections, mounted onto Superfrost Plus slides (Thermo Fisher Scientific), and then stored at −80 °C until further use. Slides were fixed in 10% neutral-buffered formalin for 15 minutes at 4 °C and were dehydrated in gradient ethanol solution. Sections were used for manual RNAscope assay using RNAscope detection kit (Advanced Cell Diagnostics). Probes were ordered from Advanced Cell Diagnostics using the custom probe design service.

#### Immunofluorescence staining

As described previously (23), adrenal glands were sliced into 6-µm thick sections using the cryostat microtome (Thermo Scientific) and sections were place in ice-cold phosphate-buffered saline (PBS). Tissue sections were permeabilized with PBS including 0.1% Tween detergent for 5 minutes. Tissues were blocked with 5% goat serum and then incubated with the 1:100 diluted CYP11B1 (RRID: AB_3076259) and CYP11B2 (RRID: AB_3076258) antibodies (Gomez-Sanchez) in 1% bovine serum albumin (w/v) in PBS-T overnight at 4 °C. Sections were washed with PBS 3 times and incubated with 1:500 diluted antimouse (RRID: AB_2338902)and antirabbit (RRID: AB_2338078) Alexa 647 (Jackson), respectively. For nuclear counterstaining, 20 µM Hoechst 33342 (Invitrogen H3570) was used. After washing 3 times, coverslips were mounted onto the slides with mounting medium FluorSave (Millipore 345 789).

#### Western blot

Western blots were performed with mouse adrenal and human NCI-H295R cell total protein obtained by homogenization in radioimmunoprecipitation assay buffer containing 50 mM Tris-HCl (pH 7.4), 150 mM NaCl, 1% NP-40, 0.5% deoxycholate acid sodium salt supplemented with phenylmethylsulfonyl fluoride (PMSF), and protease inhibitor cocktail (Roche, 04 693 159 001). Total proteins were separated by electrophoresis in 10% sodium dodecyl sulfate–polyacrylamide gels and transferred to nitrocellulose membranes (Bio-Rad). After blocking with 5% powdered milk in 0.1% TBS-Tween buffer, membranes were incubated overnight at 4 °C with primary antibodies (Table 1). After extensive washing with TBS-Tween buffer, membranes were further incubated with appropriate secondary antibodies (see Table 1) for 1 hour at room temperature and finally were exposed to chemiluminescent substrate. Protein signals were detected on an LAS-4000 Luminescent Image Analyzer and the signals were quantified with Image J. The expression of the proteins of interest was normalized to the abundance of β-actin and β-tubulin.

**Table 1.** Primary and secondary antibodies used for Western blot

Primary antibody	Species	Dilution	Expected size (kDa)	Company	RRID
pERK1/2	Rabbit	1:2000	42-44	Cell Signaling	AB_331646
Total ERK1/2	Rabbit	1:2000	42-44	Cell Signaling	AB_330744
StAR	Rabbit	1:1000	28	Cell Signaling	AB_10889737
β-Actin	Mouse	1:500	43	Merck Millipore	AB_2571580
β-Tubulin	Rabbit	1:1000	52	Cell Signaling	AB_823664
**Secondary antibody**		**Dilution**		**Company**	**RRID**
Antimouse (HRP)		1:5000		Cytiva	AB_772210
Antirabbit (HRP conjugated)		1:5000		Cytiva	AB_772191

Abbreviations: ERK, extracellularly regulated kinase; HRP, horseradish peroxidase.

#### Statistics

Data are represented as mean ± SEM. Significance was tested using a one-way analysis of variance with Bonferroni post hoc correction. *P* values smaller than .05 were considered statistically significant.

Software used for analysis were Fiji/ImageJ and GraphPad Prism 5 (GraphPad Software).

## Results

### Klotho Modulates *CYP11B2* Expression in Human NCI-H295R Adrenocortical Cells in Response to Angiotensin II

**Figure 2.**
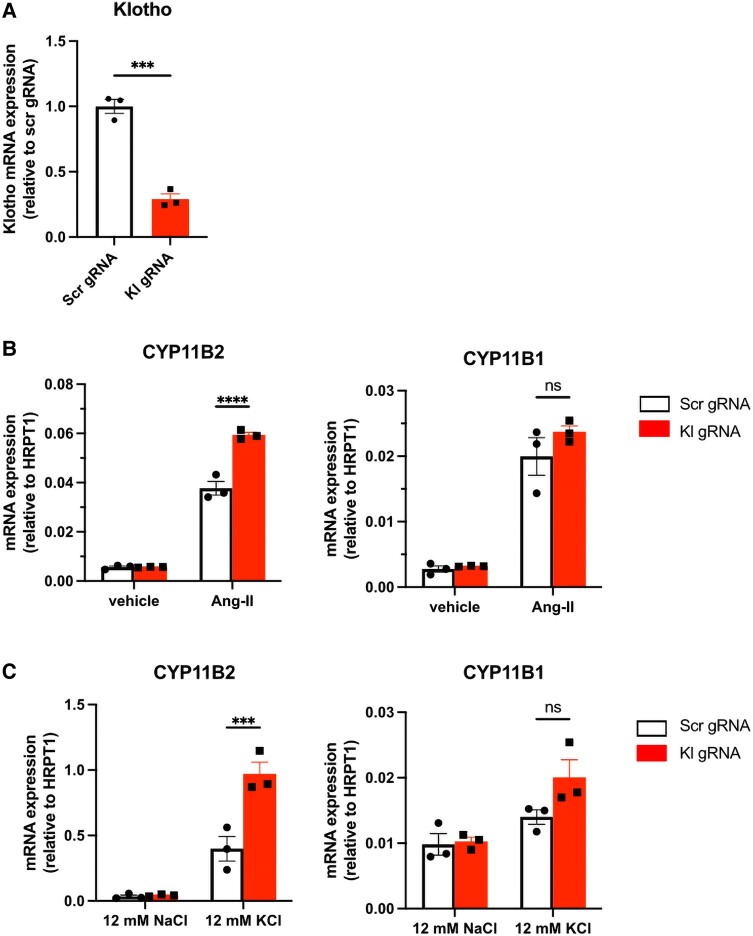
*CYP11B1* and *CYP11B2* messenger RNA (mRNA) expression level in Klotho-depleted NCI-H295R cells. A, Endogenous *Klotho* mRNA expression in Kl guide RNA (gRNA)-transfected NCI-H295R. B, *CYP11B2* and *CYP11B1* mRNA expression following 6 hours of angiotensin II stimulation in Kl gRNA-transfected NCI-H295R. C, *CYP11B2* and *CYP11B1* mRNA expression following 6 hours of 12 mM KCl stimulation in NCI-H295R cells transfected with Kl gRNAs leading to Klotho deficiency. mRNA expression levels of genes were normalized to housekeeping gene hypoxanthine guanine phosphoribosyltransferase (*HPRT*). Data are presented as mean ± SEM (n = 3 per group). Stars represent significance vs corresponding control. NS, not significant; ***P less than or equal to .001; ****P less than or equal to .0001.

NCI-H295R cells are a suitable model for the study of human adrenal cortex steroidogenesis signaling pathways. To study whether endogenous Klotho depletion affects regulation of adrenal steroidogenesis in response to Ang II, we modulated *Klotho* gene expression using CRISPR-Cas-9 technology in NCI-H295R cells. Thereby, endogenous *Klotho* gene was diminished up to 70% in Kl gRNA-transfected cells as shown in Fig. 2A. Klotho-depleted NCI-H295R cells displayed no change of *CYP11B2* gene expression at baseline. However, following both Ang II and high potassium stimulation, Klotho-depleted NCI-H295R cells showed significantly higher *CYP11B2* gene expression compared to control cells with no change in *CYP11B1* expression (Fig. 2B and 2C), suggesting a specific role of adrenal-derived *Klotho* on aldosterone synthesis.

**Figure 3.**
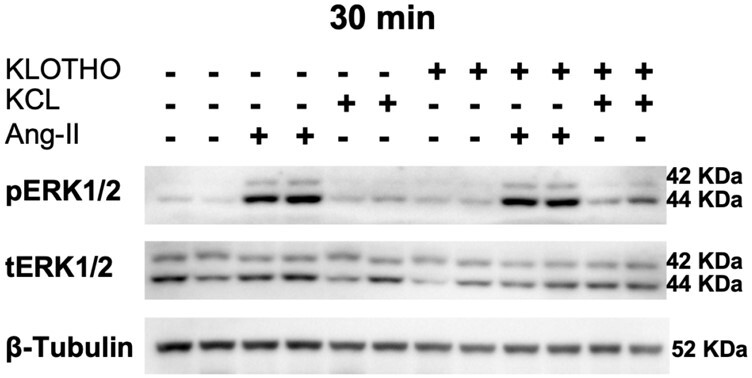
Erk1/2 phosphorylation on Klotho treatment and potassium or angiotensin II (Ang II) stimulation in NCI-H295R cells. Representative Western blot of ERK1/2 phosphorylation on overnight Klotho-treated NCI-H295R cells following 30 minutes of either Ang II or potassium (KCl) stimulation. β-Tubulin has been used as loading control.

NCI-H295R cells have been shown to respond to Ang II, triggering extracellularly regulated kinase (ERK1/2) phosphorylation in a transient fashion (24). In primary cultures of rat adrenal glomerulosa cells, Ang II activates the ERK1/2 signaling pathways and results in an increase in *STAR* expression, steroidogenic enzymes, and steroid synthesis (25, 26). To explore whether recombinant Klotho protein might affect the ERK1/2 signaling pathway, NCI-H295R cells were incubated with and without Klotho following 30 minutes of either Ang II or potassium stimulation, the main stimulators of aldosterone production (27). As shown in Fig. 3, Klotho did not affect the acute response of NCI-H295R cells to Ang II or potassium regarding ERK1/2 phosphorylation, indicating only minor, if any, effects of Klotho on ERK1/2 signaling under acute stimulation of Ang II and potassium in NCI-H295R cells.

### Recombinant Klotho Downregulates *CYP11B2* and Regulatory Transcription Factors Expression in Primary Adrenal Cells

**Figure 4.**
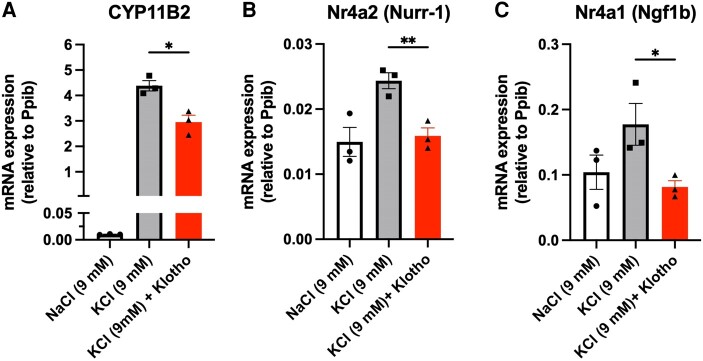
Ex vivo mouse adrenal *Cyp11b1*, *Cyp11b2*, *Nr4a1*, and *Nr4a2* expression level on Klotho treatment and potassium stimulation. A, Mouse adrenal *Cyp11B2* messenger RNA (mRNA) expression level on overnight recombinant Klotho treatment and 6 hours’ potassium (KCl) stimulation in female C57BL/6 wild-type mice. NaCl group is considered as control for KCl. B, Mouse adrenal *Nr4a2* and C, *Nr4a1* mRNA expression level on overnight recombinant Klotho treatment and 6 hours’ KCl stimulation in female C57BL/6 wild-type mice. mRNA expression levels of genes were normalized to housekeeping gene peptidylpropyl isomerase B (*Ppib*). Data are presented as mean ± SEM (n = 3-4 per group). Stars represent significance vs corresponding control. *P less than or equal to .05; **P less than or equal to .01.

To further investigate Klotho’s role in the regulation of aldosterone synthesis, we incubated mouse primary adrenal cells with potassium in the presence and absence of recombinant Klotho protein. As expected, potassium induced *CYP11B2* gene expression up to several-fold in comparison to control cells, whereas the recombinant Klotho treatment significantly decreased *CYP11B2* expression, suggesting a negative regulatory role of Klotho on *CYP11B2* expression (Fig. 4A). The inhibitory role of Klotho on *CYP11B2* expression was further confirmed by decreased expression of *Nr4a1* and *Nr4a2* on potassium stimulation (Fig. 4B and 4C).

### Zona Glomerulosa–Specific Reduction of Klotho Expression In Vivo

The Cre/loxP system has been used extensively for conditional gene manipulation in mice. As a marker of Cre-ER activity, we used a double-fluorescent Cre reporter mouse expressing membrane-targeted tandem dimer Tomato (mT) prior to Cre-mediated excision and membrane-targeted green fluorescent protein (mG) following excision (28). To specifically target CYP11B2-positive cells, we used inducible *CYP11B2^+/Cre^*-*mTmG* mice. Specifically, 8-week-old female mice received either vehicle or tamoxifen (TMX, 2 mg/kg/day) for 5 days. Two weeks following TMX treatment, we observed robust and specific marking of zG cells with green fluorescence due to TMX-induced Cre activity (Fig. 1A). To deplete Klotho in CYP11B2-expressing cells, *Klotho^fl/fl^* and *Klotho^Wt^* were crossed to *Cyp11b2^+/Cre-ER^-mTmG* to produce *Cyp11b2^+/Cer-ER^*–*Klotho^fl/fl^-mTmG* (zG-Kl-KO) and *Cyp11b2^+/Cer-ER^*–*Klotho^Wt^-mTmG* (zG-Cre) animals, respectively. zG-Kl-KO and zG-Cre mice were treated with TMX, as discussed earlier, and examined for Klotho expression in the adrenal gland by in situ hybridization. Klotho was detectable in zG cells of zG-Cre mice (Fig. 1B) and was diminished by about 65% in zG-Kl-KO mice compared to zG-Cre animals as evidenced by fluorescence intensity measurement (Fig. 1C). We further confirmed that the expression of Klotho remained unchanged in the kidney of zG-Kl-KO mice compared to zG-Cre (data not shown), suggesting tissue-specific Klotho depletion in our inducible mouse model.

### Zona Glomerulosa–Depleted Klotho in Female Mice Increased *CYP11B2* and Regulatory Transcription Factors Expression Without Effects on Systemic Aldosterone Levels

**Figure 5.**
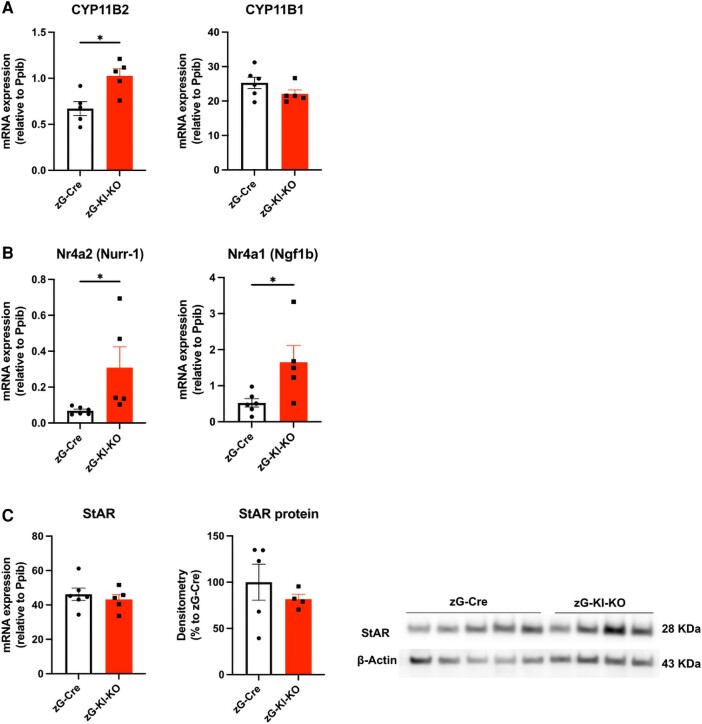
Adrenal *Cyp11b1*, *Cyp11b2*, *Nr4a1*, and *Nr4a2 and StAR* expression level in zG-Kl-KO mice. A, Adrenal messenger RNA (mRNA) expression of *Cyp11b2* and *Cyp11b1*; B, *Nr4a1* and *Nr4a2*; and C, *StAR* mRNA and protein levels in 12-week-old zG-Cre and zG-Kl-KO female mice after induction of Cre activity. mRNA expression levels of genes were normalized to housekeeping gene peptidylpropyl isomerase B (*Ppib*). Data are presented as mean ± SEM (n = 8 per group). Stars represent significance vs corresponding control; *P less than or equal to .05.

To explore the effect of zG-specific Klotho depletion on aldosterone production, we assessed the expression of 11β-hydroxylase (*CYP11B1* gene), aldosterone synthase (*CYP11B2* gene), and 2 transcription factors, nerve growth factor-induced clone B NGFIB (*Nr4a1*) and Nur-related factor 1 Nurr1 (*Nr4a2*) that affect *CYP11B2* expression. *Nr4a2* has been reported to upregulate aldosterone synthase expression in NCI-H295R cells and proposed to have clinical importance, since its expression levels are upregulated in aldosterone-producing adenomas and correlate with aldosterone synthase expression levels (29, 30). Notably, expression of *CYP11B2*, *Nr4a1*, and *Nr4a2* were each increased in zG-Kl-KO female mice, but not in males, comparing to the age-matched zG-Cre group (Fig. 5A and 5B; male data not shown), suggesting activation of the mineralocorticoid gene pathway activation, whereas *CYP11B1* and *StAR* expressions were not affected.

**Figure 6.**
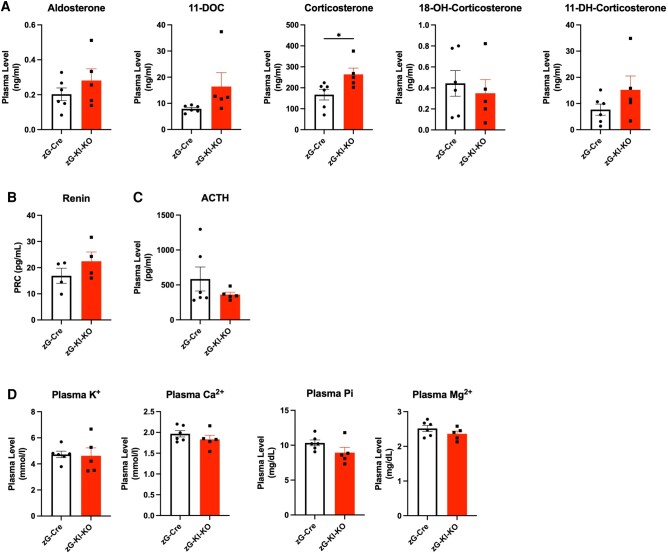
Hormonal workup and serum electrolytes in zG-Kl-KO mice. A, Plasma levels of steroid hormones including aldosterone, 11-deoxycorticosterone (11-DOC), corticosterone, 18 hydroxy-corticosterone (18OH), 11-dehydro-corticosterone (11-DH); B, plasma renin; and C, adrenocorticotropin (ACTH) concentration in 12-week-old zG-Cre and zG-Kl-KO female mice after Cre activation. D, Plasma values of potassium (K^+^), calcium (Ca^2+^), phosphate (Pi), and magnesium (Mg^2+^) of female zG-Kl-KO mice after Cre activity induction. Stars represent significance vs corresponding control; *P less than or equal to .05.

Next, we assessed the response of steroids in the mineralocorticoid pathway to zG-specific Klotho ablation. While hyperaldosteronism has been described in various Klotho-deficient mouse models in the literature (14-16), we observed no change in plasma aldosterone levels (or its precursors) in zG-Kl-KO mice compared to controls, suggesting a minor effect of zG-specific Klotho on aldosterone production (Fig. 6A). In line with unchanged aldosterone levels, we detected no significant change in either plasma renin concentration (Fig. 6B) or renal renin expression (data not shown) in the zG-Kl-KO group compared to control mice. As a potential indicator of stress, a significant increase in plasma corticosterone levels was noted in zG-Kl-KO mice (see Fig. 6A). Since relative change of plasma corticosterone concentration is regulated by the hypothalamic-pituitary-adrenal axis in response to stress (31, 32), we checked the plasma ACTH level in zG-Kl-KO mice. Despite increased plasma ACTH levels observed in Kl-deficient mice (15), plasma ACTH levels were comparable between zG-Kl-KO and control mice (Fig. 6C). Moreover, no differences in plasma potassium levels were evident between the genotypes as well as other measured electrolytes (Ca^++^, Pi, Mg^++^; Fig. 6D).

## Discussion

In the present study, we examined the influence of Klotho, a protein implicated in a variety of conditions including electrolyte balance, on aldosterone synthase expression within the context of adrenal physiology. Using different experimental approaches, we have delineated a novel role for zG-derived Klotho in modulating aldosterone synthase (*CYP11B2*) expression in young female mice. Our findings suggest that Klotho directly downregulates *CYP11B2* and its regulatory transcription factors, specifically Nr4a1 and Nr4a2, without overtly affecting plasma aldosterone levels.

*Klotho* is a gene that was initially described in the context of aging (1-4). It is primarily expressed in the kidney, where it regulates fluid and electrolyte homeostasis. The interplay between Klotho and adrenal steroidogenesis has been under investigation, given its potential implications for pathophysiological states such as primary aldosteronism (14-16). Our data provide evidence supporting the hypothesis that Klotho acts as a negative regulator of *CYP11B2* gene expression. This regulatory role is substantiated by our in vitro experiments, where Klotho depletion using CRISPR-Cas-9 technology resulted in elevated *CYP11B2* expression following Ang II stimulation in adrenocortical NCI-H295R cells. The specificity of this regulation was further emphasized by the lack of change in *CYP11B1* expression.

Notably, the ERK1/2 signaling pathway, a well-established mediator of Ang II–induced aldosterone synthesis, did not appear to be influenced by Klotho under acute stimulation conditions. This finding contrasts with the existing literature that links Klotho with various signaling pathways, suggesting that Klotho's regulatory mechanisms may be pathway-selective or context-dependent.

Our ex vivo and in vivo experiments further substantiated these in vitro data. We made use of a novel mouse model for zG-specific reduction of Klotho, achieved via a Cre/loxP approach, which led to an upregulation of *CYP11B2* and transcription factors *Nr4a1* and *Nr4a2*, key players in mineralocorticoid synthesis (33). This upregulation was evident in female but not in male mice. Intriguingly, despite the molecular evidence of enhanced aldosterone synthesis pathway activity, plasma aldosterone levels remained unchanged in zG-specific Klotho-depleted mice. Thereby, our results suggest that while zG-specific Klotho depletion leads to molecular changes indicative of stimulated aldosterone synthesis pathways, these alterations are not sufficient to induce a state of hyperaldosteronism. There are a number of potential reasons to explain this inconsistency: First, it may be attributed to the subtlety of in vivo changes, which, although sufficient to drive gene expression, do not affect systemic hormone profiles. Second, in vivo compensatory mechanisms may be in place, which could include hormonal feedback loops and intraglandular homeostasis that effectively dampen the increase in local gene expression, thus maintaining systemic hormonal balance. Third, the utilization of young mice in a physiological state of equilibrium—absent any external stressors such as a low-sodium diet or pharmacological provocations—raises the possibility of a homeostatic capacity to buffer genetic perturbations. Fourth, the developmental stage of the animals may reflect a window of heightened compensatory efficiency, preventing the full phenotypic expression of Klotho's influence on aldosterone levels. Finally, sex-dependent differences might have played a role that can readily affect an adrenal phenotype.

The degree of in vivo Klotho modulation might be a crucial determinant of the phenotypic outcome. In our study, a reduction in Klotho expression did not reach that of a complete KO, which could be a critical threshold for the manifestation of a systemic phenotype. Indeed, the requirement for a more substantial or complete depletion of Klotho to elicit pronounced changes highlights the possibility that adrenal Klotho may exert its effects in a dose-dependent manner, where only severe reductions result in noticeable systemic alterations. Furthermore, the specificity of Klotho's actions within the adrenal context may delineate a model where its role is confined to fine-tuned local regulation rather than influencing systemic hormonal levels. The sensitivity and timing of systemic aldosterone measurements may also account for the absence of detectable differences. Subtle changes or transient elevations in hormone levels could have eluded our detection methods, emphasizing the need for assays with higher sensitivity or longitudinal monitoring to capture peak hormonal fluctuations. No effect on electrolyte homeostasis, however, was further evidence of a lack of hyperaldosteronism in this model.

This study also underscores the discrepancy that can arise between in vitro findings and in vivo phenotyping. While Klotho depletion in NCI-H295R cells resulted in a marked upregulation of *CYP11B2* post Ang II stimulation, the in vivo context presented a more complex scenario, possibly reflecting the influence of systemic factors and intercellular communications not present in cell cultures.

In conclusion, our data highlight the nuanced role of Klotho in adrenal physiology, arguing for a threshold-dependent, homeostatically balanced, and potentially localized regulatory mechanism. This work reinforces the notion that adrenal steroidogenesis is part of a tightly regulated network, with some resistance to singular genetic manipulations. The findings herein provide a foundation for future studies, which should include older animals, male mice, varied physiological stressors, and a broader scope of genetic modifications to fully elucidate Klotho's place within zG physiology.

## Data Availability

Original data generated and analyzed during this study are included in this published article or in the data repositories listed in “References.”

## Abbreviations

ACTH: adrenocorticotropin
Ang II: angiotensin II
ERK: extracellularly regulated kinase
FGF: fibroblast growth factor
gRNA: guide RNA
KO: knockout
mRNA: messenger RNA
PBS: phosphate-buffered saline
RAAS: renin-angiotensin-aldosterone system
TMX: tamoxifen
zG: zona glomerulosa
